# Aerosols as a source of dissolved black carbon to the ocean

**DOI:** 10.1038/s41467-017-00437-3

**Published:** 2017-09-11

**Authors:** Hongyan Bao, Jutta Niggemann, Li Luo, Thorsten Dittmar, Shuh-Ji Kao

**Affiliations:** 1 State Key Laboratory of Marine Environmental Science, Xiamen University, Xiamen, 361102 China; 2 Collage of Ocean and Earth Sciences, Xiamen University, Xiamen, 361102 China; 3 Research Group for Marine Geochemistry (ICBM-MPI Bridging Group), Institute for Chemistry and Biology of the Marine Environment (ICBM), Carl von Ossietzky University of Oldenburg, Carl-von-Ossietzky-Str. 9-11, 26129 Oldenburg, Germany

## Abstract

Dissolved black carbon (DBC) is the largest known slow-cycling organic carbon pool in the world’s oceans. Atmospheric deposition could significantly contribute to the oceanic DBC pool, but respective information is lacking. Here we estimate that, during the dust outbreak season, the atmospheric dry deposition of water-soluble black carbon (WSBC) is ~ 40% of the riverine input to the China coastal seas. The molecular composition of atmospheric WSBC determined by ultrahigh-resolution mass spectrometry, reveals similar soil-derived sources as for riverine discharge. WSBC is significantly positively correlated with water-soluble organic carbon (WSOC) in marine aerosols, and water-soluble black carbon contributes on average 2.8 ± 0.65% to the total WSOC. Based on this relationship, the global atmospheric deposition of DBC to the ocean is estimated to be 1.8 ± 0.83 Tg yr^−1^. Anticipated future changes in biomass burning and dust mobilization might increase these numbers, with consequences for regional ecosystems and global carbon reservoirs.

## Abstract (web summary)

The contribution of atmospheric deposition to the oceanic dissolved black carbon pool (DBC) is unclear. Here, the authors show that water-soluble black carbon is positively correlated with water-soluble organic carbon in marine aerosols, and that atmospheric deposition is a significant source of oceanic DBC.

## Introduction

Biomass and fossil fuel burning emit a continuum of thermally altered organic matter (black carbon, BC) to aquatic and atmospheric environments. Owing to differences in burning temperatures, the carbon released by burning includes highly refractory compounds^1^, as well as soluble and labile (fast-cycling) organic matter (e.g., anhydrosugars and methoxyphenols)^2, 3^. Dissolved BC (DBC) is by far the largest known refractory dissolved organic carbon (DOC) pool in the ocean^4^. It can persist in the open ocean for tens of thousands of years^5–7^, having a much slower average turnover rate than BC in soils^8^. Therefore, the cycling of DBC in the ocean is a crucial component of the global carbon budget, and it is important to constrain the sources and sinks of oceanic DBC.

Every year, rivers deliver ~ 26 Tg of DBC derived from the dissolution of charcoal in soils to the ocean^9^, and this process represents by far the largest known source of oceanic DBC. Compared with riverine discharge, atmospheric transport is fast and efficient. Recent studies have shown that atmospheric deposition is one of the major pathways by which BC reaches the ocean^10, 11^. Oxidation of BC can enhance its water solubility in aerosols and rainwater^12, 13^. Considering the large amount of BC emitted by combustion sources to the atmosphere globally (4.3–22 Tg yr^−1^)^14^, the oxidation of BC is a potentially major source of DBC to the ocean. On the other hand, 420–480 Tg of dust, which contains a substantial fraction of soil organic matter (OM)^15, 16^, is deposited in the world’s oceans annually^17, 18^, making atmospheric deposition of dust another potentially important pathway for the delivery of DBC to the ocean. Recent studies have reported condensed aromatic compounds in dissolved organic matter in aerosols^19, 20^, hailstones^21^, coastal rainwater^22^, bulk deposition^23^, and snow^24, 25^, demonstrating the presence of DBC in atmospheric deposition. However, it is unknown how much of this DBC is eventually deposited in the oceans.

Asian dust, which mainly originates from northern China, Mongolia and western China, is the second largest dust source on Earth (accounting for 10–25% of global dust emissions^26^) and the largest dust source to the North Pacific Ocean. The dust storm outbreaks in spring, from February to May, disperse across the North Pacific, sometimes even reaching North America^26^. A significant amount of dust is deposited in the North Pacific Ocean (31–92 Tg yr^−1^)^17^. Moreover, North China relies heavily on energy generated by biomass and coal burning, which emits BC^27^. Coupled with hotspots of dust and BC emissions in North China, the downwind North Pacific Ocean may receive significant amounts of water-soluble black carbon (WSBC) from atmospheric deposition. To study the contribution of atmospheric deposition to the oceanic DBC pool, we carried out an aerosol sampling campaign in spring 2015 that extended from the China coastal seas (the Yellow Sea (YS) and the East China Sea (ECS)) to the northwestern Pacific Ocean.

We quantified water-soluble organic carbon (WSOC), solid-phase extractable WSOC (SPE-WSOC), and WSBC in the aerosol samples. WSBC was determined in the SPE fraction by the molecular benzenepolycarboxylic acids (BPCAs) method, which is currently the most unambiguous method for DBC quantification^28^. The respective DBC fraction has been quantified in rivers and throughout the open ocean water column^4, 9, 29^, making the results of our study directly comparable to these previously characterized DBC pools. For riverine DOM (International humic substances society (IHSS) natural organic matter reference material), the SPE extraction of DBC is quantitative (100%) when the fraction quantified with the BPCAs method is considered. WSBC is defined as the BC that can be dissolved in ultrapure water, as the majority (~ 70–80%) of atmospheric organic carbon (OC) deposited in the ocean is in the form of wet deposition^10, 30^, whereas OC is mainly associated with freshwater. In addition, the method is the most established technique for the extraction of WSOC from aerosol particles, making our results comparable to those of other studies. Ultrahigh-resolution Fourier transform ion cyclotron resonance mass spectrometry (FT-ICR-MS) was applied to provide molecular compositional information for SPE-WSOC and infer the sources of WSBC. FT-ICR-MS is by far the most powerful mass spectrometry method for molecular analysis, as it provides a broad and very detailed overview of dissolved organic matter (DOM) molecular composition. Molecular-level DBC and FT-ICR-MS analyses are complementary; they provide comprehensive information on the quality and quantity of DBC in oceanic aerosols. We find that the concentration of WSBC in marine aerosols is highly positively correlated with the concentration of WSOC. Further, we estimate that the atmospheric deposition of WSBC in the global ocean is 1.8 ± 0.83 Tg yr^−1^. We are the first to show that atmospheric deposition is a significant source of oceanic DBC, the most refractory fraction of DOC identified to date in the global ocean.

## Results

### Sample classification

**Fig. 1:**
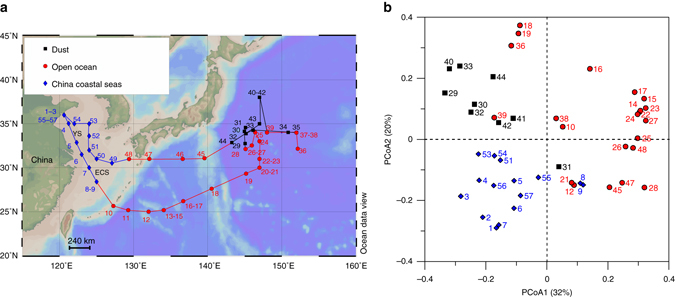
Sampling map and the result of principal coordinates analysis. **a** Sampling map. Samples were classified according to the sampling locations, satellite images and backward trajectories (Supplementary Figs. 1 and 2). **b** Principal coordinates analysis (PCoA) based on the Bray–Curtis dissimilarity distance calculated from the intensity normalized molecular composition of each sample. The values of the Bray–Curtis dissimilarity (which ranges from 0, meaning completely identical, to 1, meaning completely different) for the samples ranged from 0.12 to 0.74. The largest difference was found between the dust and the open ocean aerosols. Sample groups classified by their locations and backward trajectories were highly consistent with PCoA clusters, with only a few exceptions (Fig. 1b; Methods). *YS* Yellow Sea, *ECS* East China Sea

During the sampling campaign, we observed various types of aerosols, including pollution mixed with dust, dust and open ocean aerosols. Samples were further separated into three groups according to their sampling locations, backward trajectories and satellite images, namely, China coastal seas, dust and open ocean aerosols (Fig. 1, Supplementary Fig. 1 and Supplementary Fig. 2; also see the Methods section). Interestingly, principal coordinates analysis (PCoA) based on the molecular composition of the SPE-WSOC (thousands of molecular formulas) clearly separated these groups of aerosol samples, and the results were fully consistent with the origins of the aerosols based on sampling location and backward trajectories (Fig. 1 and Methods).

### WSOC and WSBC concentrations

**Fig. 2:**
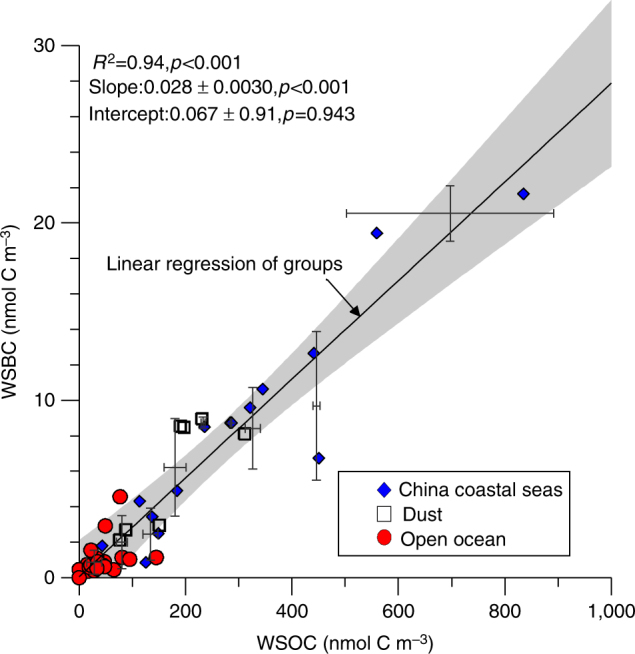
Relationship between water soluble black carbon and water soluble organic carbon. Error bars show the 1 s.d. of the average values for each concentration group. The statistics are for the regression of the average values. The *grey area* shows the 95% confidence interval of the linear regression

WSOC and WSBC concentrations varied by two orders of magnitude and ranged from 6.8 to 835 nmol C m^−3^ and 0.10 to 22 nmol C m^−3^, respectively (Fig. 2 and Supplementary Data 1). Concentrations decreased from the China coastal seas to the open ocean aerosols. The observed WSOC concentrations in the open ocean aerosols (41 ± 32 nmol C m^−3^, 1 s.d.) were close to those previously reported in the northwestern Pacific during summer (28–45 nmol C m^−3^)^31^. The WSOC and WSBC concentrations of the dust aerosols in the open ocean were on average five times higher than those of open ocean non-dust aerosols (Fig. 2), reflecting the important role of dust aerosols in transporting WSBC to the ocean. Higher WSOC concentrations in dust aerosols from North China relative to air masses from the adjacent ocean have been reported previously, and the dust WSOC concentrations in our study fall in a similar range^32^.

The fraction of WSBC in WSOC (i.e., the WSBC/WSOC ratio) in the aerosols ranged from 0.0068 to 0.070 (mean: 0.027). The differences in WSBC/WSOC ratios among the three groups of aerosols were insignificant (*t*-test, *p* > 0.05). Even though the molecular composition of WSOC showed huge dissimilarities among samples (see the caption of Fig. 1), the concentration of WSBC was highly significantly and positively related to WSOC for all aerosol samples (slope = 0.027 ± 0.0020, 1 s.d., *R* ^2^ = 0.87, *p* < 0.001, *n* = 44). Because the WSOC and WSBC concentrations were not normally distributed, we grouped the samples into nine groups according to their WSOC concentrations for further analysis. The slope of the grouped WSBC-WSOC relationship is 0.028 ± 0.0065 (95% confidence interval, *R* ^2^ = 0.94, *p* < 0.001; Fig. 2), which is highly consistent with the original dataset. Based on this result, WSBC accounts for 2.8 ± 0.65% (95% confidence interval) of the total WSOC in aerosols.

### Molecular composition revealed by FT-ICR-MS

Approximately 2900–6800 formulas in each sample and 9690 unique formulas in total were assigned. A summary of the general molecular composition is provided in Supplementary Table 1. The masses of the assigned formulas ranged primarily from 150 to 500 Da (Supplementary Fig. 3), and the intensity weighted mass values were 316–393 Da (Supplementary Table 1). Molecular formulas that included only C, H and O (CHO) were the most abundant compound group in almost all of the samples (30–67%), followed by CHON compounds (composed of C, H, O, and N; 12–35%). China coastal seas samples had a much higher fraction of CHOS formulas (composed of C, H, O, and S; mean: 32%) than both dust and open ocean aerosols (mean: 14 and 16%, respectively; Supplementary Table 1).

**Fig. 3:**
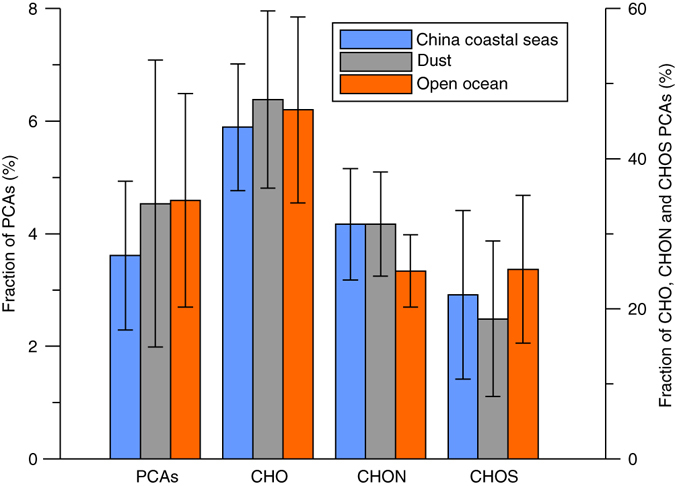
The fraction of polycyclic aromatic compounds in the different types of aerosols. The *left vertical axis* refers to the fraction of polycyclic aromatic compounds (PCAs, % of total signal intensity), whereas the *right vertical axis* indicates the fraction of different PCAs (CHO, CHON, and CHOS) in different aerosol types. Error bars show 1 s.d. of the average values for each sample group. CHO: compounds composed of C, H, and O; CHON: compounds composed of C, H, O, and N; CHOS: compounds composed of C, H, O, and S

Compounds with a modified aromaticity index (AI_mod_) of ≥ 0.67 are considered to be polycyclic aromatic compounds (PCAs)^33^, which include BC molecules. We detected 138–414 (596 in total) PCAs in each sample, and these compounds accounted for 1.3–10% of the total signal intensity of each sample (Fig. 3 and Supplementary Fig. 4). Similar relative abundances of the PCAs have been reported for a variety of atmospheric deposition types, including coastal rainwater (2%)^22^, bulk deposition in the coastal zone of Finland (number of formulas, ~ 5%)^23^, snowpack in Antarctica (1–9%)^25^, snow on the Greenland ice sheet (12.6%)^24^ and aerosols in New York and Virginia (1–4%)^19^. The semi-quantitative information derived from FT-ICR-MS analysis was consistent with the quantitative DBC analyses, as indicated by the significant correlation between the WSBC/WSOC ratio and the relative abundance of PCAs molecular formulas (*p* < 0.01, *n* = 44; Supplementary Fig. 4).

PCAs are mainly CHO compounds (intensity normalized, 46 ± 11%, *n* = 46), followed by CHON (28 ± 7%, *n* = 46; Fig. 3). The abundances of CHOS PCAs fractions (22 ± 10%, *n* = 46) are similar to those of CHON. Other PCAs (including CHONS (composed of C, H, O, N, and S), CHOP (composed of C, H, O, and P), and CHONP (composed of C, H, O, N, and P)) made up only a small fraction (3 ± 2%, *n* = 46) (Supplementary Table 2).

## Discussion

Considering that the composition of atmospheric WSOC was highly variable, the observed significant correlation between WSBC and WSOC in natural aerosol samples was unexpected. This significant correlation suggests that WSBC and WSOC might be released by similar processes^9^. Possible sources of WSBC are the dissolution of soil particles, the oxidation of soot^13^ and seawater derived from sea spray^34^. Soil organic matter is present both in dust and in biomass burning aerosols^15, 35, 36^. In biomass burning aerosols, due to the turbulence-induced re-suspension of soil particles, soil OM can contribute up to 27% of WSOC^35^, whereas soot can be derived from biomass and fossil fuel burning. Secondary reactions in the atmosphere can produce aromatic compounds, e.g., brown carbon;^37^ however, these compounds are less condensed (AI_mod_ < 0.5) than DBC compounds and do not contribute to the BC fraction quantified by the BPCAs method. The much higher WSBC concentration in the China coastal seas and the dust aerosols compared with the open ocean aerosols suggests that sea spray was not the dominant source of WSBC.

The composition of the PCAs can further assist in deciphering the potential sources of WSBC in the marine atmosphere (Fig. 3). Burning of forest landscapes normally generates N-depleted DBC^38, 39^, and the heteroatom- (N- and S-) containing PCAs may be related to anthropogenic activities, e.g., agriculture^40^, or the burning of S-containing fuel. Secondary reactions of volatile organic compounds with SO_2_ and/or NO_X_ in the atmosphere may also produce aromatic organosulfates^41^. However, whether this process can produce highly condensed, S-containing PCAs has not been determined so far. The differences in the composition of the PCAs among different types of aerosols are mostly insignificant (t-test, *p* > 0.05), except that a slightly higher (by 5%, on average) fraction of CHON PCAs was noted in China coastal seas and the dust aerosols than the open ocean aerosols (Fig. 3 and Supplementary Table 2), which is consistent with their proximity to terrestrial environments and agricultural activities. Dissolution from soil particles is a major source of riverine DBC^9^. By comparing the PCAs of aerosols and river water samples (based on an unpublished dataset of 44 samples collected from two rivers; see the Methods for sampling information), we found that 86 ± 5% (1 s.d.) of the PCAs in aerosols can be found in rivers (defined as PCAar), and those PCAar compounds comprise 69 ± 6% (1 s.d.) of the total PCAs in the riverine samples. This finding suggests that the sources of DBC in marine aerosols are largely similar to those of riverine DBC, i.e., dissolution from soil^9^, consistent with the molecular composition of the PCAs in marine aerosols (which are mainly composed of CHO and CHON compounds). A previous study of radiocarbon in WSOC in the airmass outflow of northern China also indicated that non-fossil sources (60–90%) dominated total WSOC^32^. However, the presence of CHOS PCAs may indicate some contribution from fossil fuel burning. Heteroatom- (N- and S-) containing PCAs were also detected in other atmospheric depositions, including marine aerosols in the Atlantic Ocean^20^, coastal rainwater^22^, and surface snow on the East Antarctic ice sheet^25^, suggesting the distribution of anthropogenic DBC in atmospheric deposition, even in remote open ocean areas.

Atmospheric processes (e.g., photochemical degradation^2, 42^), contributions from other sources (e.g., peptides potentially derived from ocean spray)^43^ or the formation of secondary organic aerosols (e.g., isoprene-derived SOA)^44^ may affect the WSBC/WSOC ratio in atmospheric deposition, which also explains the lower fraction of WSBC in atmospheric WSOC compared with DBC in riverine DOC^9^. Aerosol type may not be an important factor affecting the WSBC/WSOC ratios, as the differences in the average values of WSBC/WSOC ratios are insignificant among the three groups of aerosols. The correlation of WSBC/WSOC ratios with humidity and wind speed, as well as temperature, were all insignificant (*p* > 0.05; data not shown). The consistency of the WSBC/WSOC ratio under different weather conditions suggests that these factors do not exert a significant influence on WSBC/WSOC ratios. However, to what extent other factors (e.g., aerosol size, surface/volume ratio, total OC, travel time, latitude, and radiation) might affect the fraction of WSBC in atmospheric WSOC remains unclear.

To explore whether DBC in atmospheric deposition contributes to the oceanic DBC pool, we compared the PCAs in aerosols with seawater collected in the same region (depths ranging from 5 to 4000 m; see the Methods for sampling information). Approximately 45% (intensity normalized) of the PCAs in aerosols were also found in seawater (defined as PCAas; Supplementary Table 2). Those PCAas made up 50 ± 19% (1 s.d., 8–82%, intensity normalized, *n* = 150) of the total PCAs in seawater on average, suggesting a potential contribution of atmospheric deposition to the oceanic DBC pool. The smaller fraction of PCAas in aerosols compared to PCAar might be due to an effect of salinity on the dissolution of WSBC in seawater, as suggested by previous reports of the salting-out effect of polycyclic aromatic hydrocarbons (PAHs)^45^. Compared with PCAar, PCAas had generally higher H/C ratios, lower AI_mod_, and lower masses (intensity weighted values, Supplementary Table 2), consist with an earlier study^5^. BC-water partitioning could be a possible explanation, as it has been reported that PAHs with fewer rings and lower molecular weight are more soluble in water^45^.

Applying the average WSBC concentrations of the aerosols, we estimated that the dry atmospheric deposition of WSBC in the China coastal seas (the YS and the ECS) was 0.072 ± 0.042 Gg d^−1^ during the dust outbreak period in April 2015 (see the Methods section). This atmospheric deposition was ~ 40% of two major rivers’ DBC input to that region (see the Methods section), which highlights the temporal and regional significance of atmospheric contributions to the ocean DBC pool.

To extrapolate our result to the global scale, we take advantage of the robust relationship between WSBC and WSOC observed in our aerosol samples and assume that this WSBC/WSOC ratio is representative of atmospheric deposition in other regions. This assumption is supported by the very similar relative abundances of the PCAs detected via FT-ICR-MS in a variety of atmospheric samples representing a global range^19, 22–25^. Based on the global atmospheric WSOC deposition^30^ and assuming that all of the aerosols are finally deposited in the ocean by either dry deposition or wet deposition, we estimated that the atmospheric contribution of DBC (by both dry and wet deposition) to the global ocean is 1.8 ± 0.83 Tg yr^−1^ (the uncertainty is derived from a comprehensive assessment of error propagation, see the Methods section), with ~ 70–80% in the form of wet deposition^10, 30^. The distribution of atmospheric deposition therefore depends strongly on the regional setting and is strongly related to the wet deposition of soluble OC. The deposition of snow, which may also contain a significant fraction of DBC^24, 25^, is not included in our estimate. We further estimated that dust deposition represents 0.5 ± 0.3 Tg yr^−1^ and accounts for ~ 30% of the estimated atmospheric WSBC input to the global ocean (see the Methods for the underlying calculations).

The global WSBC flux from atmospheric deposition is much smaller than the global riverine DBC flux (26 Tg yr^−1^)^9^. Nonetheless, atmospheric WSBC deposition alone can support the oceanic DBC turnover at an average rate of ~ 6700 yr, demonstrating the significance of atmospheric deposition. Recent studies found that biomass burning also releases labile OC^2, 3^, and the entire continuum of pyrogenic organic matter and anticipated changes of atmospheric fluxes should be considered in assessments of potential impacts on ecosystems at regional and global scales. Our study is the first to show that atmospheric deposition is a significant source of oceanic DBC, one of the most refractory forms of organic matter in the ocean. Future changes in both dust and biomass burning activities may potentially affect the deposition of WSBC in marine environments^46, 47^.

## Methods

### Sample collection

Aerosol samples were collected from the China coastal seas to the northwestern Pacific Ocean from 28 March to 4 May 2015 (Fig. 1a; Ocean Data View^48^) on board the R/V Dongfanghong 2. The numbers in Fig. 1a show the sampling sequence, and most of the samples were collected during cruising. If there are two numbers placed in one location, this means that the first sample was collected during cruising and the second was collected when the ship was stopped (e.g., 20–21, 20 was collected on the way from 19 to 20, whereas 21 was collected while the ship was stopped). The aerosol sampler was placed on the top deck of the ship during cruising. Air was pumped through a filter (Whatman 41, nominal pore size: 20 µm) at a rate of ~ 1 m^3^ min^−1^, and a mean total air volume of 1140 m^3^ was applied to each filter. Here, we define the fraction of particles that is retained by this filter as representing aerosol particles. The retention efficiency for particle sizes >0.2 µm was >90% at a pumping rate of 1 m^3^ min^−1^ ^49^. All the filters were stored in ziplock bags at −20°C until analysis. Not all samples in Fig. 1a were analysed owing to limited sample size; some were used for other research purposes. In total, 46 samples were used in the present study, and their corresponding sample IDs are shown in Fig. 1b.

WSOC is defined as the OC in aerosols that can be dissolved in ultrapure water (18.2 MΩ). The extraction of WSOC followed established protocols^19^. In brief, half or a quarter of the filter was extracted with ultrapure water in an ultrasonic water bath, and the suspension was filtered through a nylon syringe filter (0.45 µm) for WSOC analysis. A defined aliquot of each filtrate was acidified to pH 2 with hydrochloric acid (32%, Merck) and concentrated by solid-phase extraction (SPE) with commercially available PPL cartridges (500 mg, Agilent, USA)^50^. Extracts were eluted from the cartridges using HPLC grade methanol (~ 6 mL, exact volume determined by weight). The extraction efficiency of DOC was determined by drying 150 µL of extract in an oven at 50°C overnight. Dry extracts were dissolved in 15 mL acidified ultrapure water (pH = 2) and solid-phase extractable DOC concentration (SPE-WSOC) was determined in the solutions as outlined below. The extraction efficiency (SPE-WSOC/WSOC) was on average 46 ± 24% on a carbon basis (mean ± 1 s.d., *n* = 44).

Seawater samples were collected from the same region as the aerosol samples, at water depths ranging from 5 m to 4000 m. River water was collected from Jiulong River located in the southeastern part of China. All water samples were filtered through 47 mm GF/F filters (pre-combusted at 450 °C). A defined aliquot of the filtrate was then acidified to pH = 2 with hydrochloric acid (32%, Merck) and extracted by solid-phase extraction (SPE) with PPL following the same procedure as aerosol WSOC. The extracts were stored at −20 °C until FT-ICR-MS analysis.

*DOC analysis.* DOC was measured using a Shimadzu TOC analyser at Xiamen University. Deep sea water reference samples from the Sargasso Sea (D.A. Hansell, University of Miami, FL, USA) were used to verify the quality of the data. The analytical error was less than 5%.

*WSBC quantification.* WSBC was determined on a molecular level using the BPCAs method^28^. In brief, an aliquot of the extract (~ 5 µmol SPE-DOC) was transferred to pre-combusted (400 °C) glass ampoules and dried in an oven at 50 °C. The dried extract was dissolved in 500 µL nitric acid (65%), the ampoules were sealed and heated to 170 °C in a stainless-steel pressure bomb for 9 h. After the ampoules cooled, 450 µL was transferred into 1 mL vials. Nitric acid was evaporated in a centrifugal evaporator (RVC 2–18, Christ, Germany). Samples were then re-dissolved in 100 µL of phosphate buffer solution (Na_2_HPO_4_ × 2 H_2_O (89 mg) and NaH_2_PO_4_ × 2 H_2_O (78 mg) in 100 mL ultrapure water) and stored frozen until analysis. BPCAs were determined on a Waters ACQUITY UPLC (ultra performance liquid chromatography) system equipped with a photodiode array light-absorbance detector. BPCAs were separated on a Waters ACQUITY UPLC BEH C18 Column (2.1 × 150 mm, 1.7 µm) with an aqueous phase/methanol gradient. The aqueous phase was prepared by dissolving 1.3 g tetrabutylammonium bromide (ACS quality), 5 mmol Na_2_HPO_4_ and 5 mmol NaH_2_PO_4_, and 100 mL methanol in ultrapure water with a final volume of 1 L. The injection volume was 10 µL. BPCAs were identified according to their retention time and absorbance spectra (220–380 nm). Quantification was performed using the absorption signal at 240 nm. Total DBC concentration was calculated using a power-function relation between the most reliably quantified B5CA and B6CA and total BC concentration generated by a set of 351 samples^51^. A natural aquatic organic matter reference from IHSS was used for quality control of the analytical procedure. The standard deviation of DBC concentrations based on replicate IHSS analyses was < 5%.

*FT-ICR-MS analysis.* SPE-WSOC extracts were diluted with ultrapure water and methanol to obtain a methanol to water ratio of 1:1 and a final concentration of 10 mg C L^−1^. Ultrahigh resolution mass spectrometry analysis was performed on a solariX FT-ICR-MS (Bruker Daltonic, Germany) equipped with a 15 Tesla magnet at Oldenburg University, following the method outlined in Seidel et al.^52^. In brief, samples were infused into the electrospray source (ESI, Apollo ion source, Bruker Daltonic, Germany) in negative mode at a rate of 2 µL/min. For each run, 500 scans were accumulated in the mass range of 150–2000 Da. The spectra were mass calibrated with an internal calibration list using the Bruker Daltonics Data Analysis software package. Only peaks with a signal to noise ratio of >5 were considered for further analysis. The final number of assigned molecular formulas for each sample was between 2900 and 6800. The aromaticity index was calculated for each formula based on its elemental composition following standard procedures^33^.

*Quality assurance.* Five blank filters were processed following the same extraction and analytical procedure as the samples. The blank concentrations (average ± 1 s.d.) for WSOC, SPE-WSOC, and WSBC were 11.4 ± 2.0 µmol C (g filter)^−1^, 1.9 ± 0.8 µmol C (g filter)^−1^, and 0.03 ± 0.01 µmol C (g filter)^−1^, respectively. The filter blanks of WSOC, SPE-WSOC, and WSBC (average ± 1 s.d.) accounted for 38 ± 22%, 25 ± 17%, and 12 ± 13% of the leachate from the filters, respectively, depending on the total amount of WSOC and WSBC in the leachate. All of the parameters were blank corrected. The WSOC and WSBC concentrations measured in all of the samples were above the laboratory blank concentrations. Sample concentrations (after blank correction) less than two times the blank standard derivation are shown as zero in Fig. 2.

One possible concern was whether the tail gas from the ship affected our sampling. No significant differences in WSOC, SPE-WSOC, and WSBC concentrations as well as WSBC/WSOC ratios were observed between the open ocean samples collected while the ship was cruising and stopped (which may potentially be affected by the ship emission) (*t*-test, *p* > 0.05), which indicates that the tail gas did not affect our sampling. This was further supported by the similar molecular composition between the open ocean aerosols and samples collected while the ship was stopped. We speculate that this might be because we mainly focused on the aerosol particles, whereas the ship emissions were mainly gases (CO_2_, NO_X_) and very fine particles (<0.1 µm) (Diesch et al., 2013) that were not efficiently retained by our filter.

### Sample classification

The classification of the samples (Fig. 1) was mainly based on the sampling locations, backward trajectories (Supplementary Fig. 1) and satellite images (Supplementary Fig. 2). The classification is highly consistent with the principal coordinates analysis based on the molecular composition determined by FT-ICR-MS (Fig. 1b), with only few exceptions that can be explained by other parameters. For example, samples #8 and #9 showed larger dissimilarities to other China coastal seas samples because they were more close to the open ocean, and this is consistent with their molecular composition. Although the molecular compositions of the open ocean samples #18, #19, #36, and #39 are much closer to those of dust aerosols, they were not included as dust aerosols by the sampling locations and backward trajectories (Fig. 1b). The potential reason is that they were close to a dust-affected region, especially samples #36 and #39, which is also consistent with the slightly higher WSBC concentration compared to other open ocean aerosols (data not shown). Sample #31 is a dust aerosol sample according to the backward trajectory and WSBC concentration, but the molecular composition is similar to the background samples, which may be because #31 is affected by moderate rain, and the molecular composition is more close to the other two rain-affected samples (#28 and #45).

### Statistical analysis

We performed Bray–Curtis dissimilarity analysis based on the sum normalized signal intensities of the assigned formulas among the samples, followed by principal coordinates analysis based on the Bray–Curtis dissimilarity matrix (as implemented in the vegan package in the R computing environment^53^). The WSOC and WSBC concentrations were tested for normality using the Shapiro.test function in R^53^. As our WSOC and WSBC concentrations were not normally distributed (*p* < 0.05), the samples were grouped according to their WSOC concentrations into nine groups. The resulting average WSOC and WSBC concentrations were normally distributed, and the linear relationship between the grouped WSOC and WSBC values was then analysed. Student’s *t*-test was used to compare the differences in concentrations and molecular compositions among three groups of aerosol. Linear regression and *t*-test were performed using SPSS 13.0 for Windows (SPSS, Inc., USA).

### Estimation of WSBC deposition in the China Coastal Seas

The deposition flux of WSBC was calculated as follows:

1 $${F_{\rm{D}}} = C \times V$$

where *F*_D_ is the dry deposition flux of WSBC (mg m^−2^ d^−1^), *C* is the concentration of WSBC (ng C m^−3^), and *V* is the deposition velocity (cm s^−1^). The deposition velocity of aerosol particles can vary over three orders of magnitude for the particles size range of 0.1–100 µm^54^. Because our samples were collected during the dust outbreak season, we employed the lower dust deposition velocity in the China coastal seas (1.4 cm s^−1^) as the deposition velocity (*V*) value^55^. Accordingly, the daily atmospheric dry deposition of WSBC in the YS and the ECS is estimated to range from 0.012 mg m^−2^ d^−1^ (for samples collected close to the northwestern Pacific Ocean) to 0.31 mg m^−2^ d^−1^ (for samples collected close to Qingdao city). The total dry deposition of WSBC in the YS and the ECS was calculated by multiplying the average ( ± 1 s.d.) WSBC flux (0.042 ± 0.033 mg m^−2^ d^−1^ and 0.10 ± 0.043 mg m^−2^ d^−1^ for the YS and the ECS, respectively; five samples that were collected very close to Qingdao city were not included in the average value) by the surface area of the YS and the ECS (380,000 km^2^ and 770,000 km^2^, respectively). The resulting dry deposition of WSBC is 0.072 ± 0.042 Gg d^−1^ (1 s.d.).

The primary riverine discharges to the YS and the ECS are from the Yellow River and the Changjiang River, respectively. The daily discharges of DOC from these two rivers in April were ~ 0.030 Gg d^−1^ and ~ 2.3 Gg d^−1^, respectively^56, 57^. There is one report of the DBC/DOC ratio for the Changjiang River (derived from one sample collected close to peak discharge), and this ratio is ~ 4.1%^9^. Considering the potential variability in DBC/DOC ratios, we used the average of this value (4.1%) and the global riverine average (~ 11%) to roughly estimate the riverine discharge of DBC to the YS and the ECS^9^. Based on these assumptions, the riverine DBC discharges from the Yellow and Changjiang Rivers to the YS and the ECS in April 2015 were ~ 0.0020 Gg d^−1^ and ~ 0.17 Gg d^−1^, respectively (0.17 Gg d^−1^ in total). Considering these estimates, the atmospheric deposition to the YS and the ECS was ~ 40% of the riverine discharge by the Yellow and Changjiang Rivers.

### Estimation of the global deposition of WSBC in the ocean

The atmospheric deposition of WSBC in the global oceans was estimated as follows:

2 $${F_{{\rm{WSBC}}}} = {F_{{\rm{WSOC}}}} \times ({\rm{WSBC/WSOC}})$$

where *F*_WSBC_ is the annual atmospheric deposition of WSBC to the global ocean, and *F*_WSOC_ is the annual global atmospheric WSOC deposition to the ocean. A recent estimate of the global deposition of WSOC associated with particles is 64 Tg yr^−1^ (dry and wet)^30^, which is close to the average atmospheric deposition of 46 Tg yr^−1^ WSOC (58 Tg yr^−1^ in total, assuming 80% of OC is soluble)^10^ and 90 Tg yr^−1^ of rainwater DOC^58^. For our global estimate, we use the recent estimate of 64 Tg yr^−1^ ^30^ and assume a 40% variation. WSBC/WSOC is the ratio of WSBC to WSOC in the aerosols from the present study (2.8 ± 0.65%, 95% confidence interval). Considering these numbers, the average deposition of WSBC to the global ocean is 1.8 ± 0.83 Tg yr^−1^ (the uncertainty range is derived from the error propagation of the global soluble OC flux and WSBC/WSOC ratio).

### Estimation of dust WSBC deposition to the Global Oceans

Dust deposition of WSBC to the global oceans is estimated using the following formula:

3 $${D_{{\rm{WSBC}}}} = {F_{{\rm{Dust}}}} \times ({\rm{WSOC/}}D) \times ({\rm{WSBC/WSOC}})$$

where *D*_WSBC_ is the dust deposition of WSBC, *F*_Dust_ is the global dust deposition flux, WSOC/*D* is the ratio of WSOC in the dust particles and WSBC/WSOC is the ratio of WSBC to WSOC in dust aerosols. Global annual dust deposition was estimated to 420–480 Tg^17^ in several previous studies. We used the median value 450 Tg yr^−1^ as global dust deposition. The WSOC/*D* displays large variation in different studies, varying from ~ 2% in Asian dust samples^32^ to 5–11% in Saharan dust^36^. We used the lower value of Sahara dust (5%) and average values of Sahara and Asian dust (3.5 ± 1.5%) as the water-soluble fraction in dust aerosols. The WSBC/WSOC in dust aerosols are on average 3.1 ± 1.0% (1 s.d., *n* = 8). Therefore, the total global dust deposition of WSBC is estimated as 0.5 ± 0.3 Tg yr^−1^, where ± represents the propagated error from the standard deviation of the WSBC/WSOC ratios and the WSOC/*D* ratio of dust.

### Data availability

Data supporting Fig. 2 and Fig. 3 can be found in the Supplementary Files. Detailed molecular compositional data are available on request from T. Dittmar.

## Electronic supplementary material

Supplementary Information

Supplementary Data 1

## References

[1] Schmidt MWI, Noack AG, Osmond G (2000). Black carbon in soils and sediments: analysis, distribution, implications, and current challenges. Global Biogeochem. Cycles.

[2] Myers-Pigg AN (2016). Signatures of biomass burning aerosols in the plume of a saltmarsh wildfire in South Texas. Environ. Sci. Technol..

[3] Myers-Pigg AN (2015). Labile pyrogenic dissolved organic carbon in major Siberian Arctic rivers: implications for wildfire-stream metabolic linkages. Geophys. Res. Lett..

[4] Dittmar T, Paeng J (2009). A heat-induced molecular signature in marine dissolved organic matter. Nat. Geosci..

[5] Ziolkowski LA, Druffel ERM (2010). Aged black carbon identified in marine dissolved organic carbon. Geophys. Res. Lett..

[6] Dittmar, T. in *Biogeochemistry of Marine Dissolved Organic Matter: Second Edition* (eds. Hansell, D. A. & Carlson, C. A.) 369–388 (Elsevier Inc., 2015).

[7] Coppola AI, Druffel ERM (2016). Cycling of black carbon in the ocean. Geophys. Res. Lett..

[8] Ohlson M, Dahlberg B, Økland T, Brown K, Halvorsen R (2009). The charcoal carbon pool in boreal forest soils. Nat. Geosci..

[9] Jaffé R (2013). Global charcoal mobilization from soils via dissolution and riverine transport to the oceans. Science.

[10] Jurado E, Dachs J, Duarte CM, Simó R (2008). Atmospheric deposition of organic and black carbon to the global oceans. Atmos. Environ..

[11] González-Gaya B (2016). High atmosphere–ocean exchange of semivolatile aromatic hydrocarbons. Nat. Geosci..

[12] Mead RN (2013). Insights into dissolved organic matter complexity in rainwater from continental and coastal storms by ultrahigh resolution Fourier transform ion cyclotron resonance mass spectrometry. Atmos. Chem. Phys..

[13] Decesari S (2002). Water soluble organic compounds formed by oxidation of soot. Atmos. Environ..

[14] Bond TC (2004). A technology-based global inventory of black and organic carbon emissions from combustion. J. Geophys. Res. Atmos.

[15] De Vicente I, Ortega-Retuerta E, Morales-Baquero R, Reche I (2012). Contribution of dust inputs to dissolved organic carbon and water transparency in Mediterranean reservoirs. Biogeosciences.

[16] Webb NP, Strong CL, Chappell A, Marx SK, Mctainsh GH (2013). Soil organic carbon enrichment of dust emissions: magnitude, mechanisms and its implications for the carbon cycle. Earth Surf. Process Landforms.

[17] Shao Y (2011). Dust cycle: an emerging core theme in Earth system science. Aeolian Res.

[18] Jickells TD (2005). Global iron connections between desert dust, ocean biogeochemistry, and climate. Science.

[19] Wozniak AS, Bauer JE, Sleighter RL, Dickhut RM, Hatcher PG (2008). Technical Note: molecular characterization of aerosol-derived water soluble organic carbon using ultrahigh resolution electrospray ionization Fourier transform ion cyclotron resonance mass spectrometry. Atmos. Chem. Phys..

[20] Wozniak AS, Willoughby AS, Gurganus SC, Hatcher PG (2014). Distinguishing molecular characteristics of aerosol water soluble organic matter from the 2011 trans-North Atlantic US GEOTRACES cruise. Atmos. Chem. Phys..

[21] Šantl-Temkiv T (2013). Hailstones: a window into the microbial and chemical inventory of a storm cloud. PLoS. ONE.

[22] Mitra S (2013). Multiproxy probing of rainwater dissolved organic matter (DOM) composition in coastal storms as a function of trajectory. Mar. Chem..

[23] Ide J (2017). Spatial variations in the molecular diversity of dissolved organic matter in water moving through a boreal forest in eastern Finland. Sci. Rep.

[24] Bhatia MP, Das SB, Longnecker K, Charette MA, Kujawinski EB (2010). Molecular characterization of dissolved organic matter associated with the Greenland ice sheet. Geochim. Cosmochim. Acta..

[25] Antony R (2014). Origin and sources of dissolved organic matter in snow on the East Antarctic ice sheet. Environ. Sci. Technol..

[26] Maher BA (2010). Global connections between aeolian dust, climate and ocean biogeochemistry at the present day and at the last glacial maximum. Earth-Science Rev.

[27] Zhang Q (2009). Asian emissions in 2006 for the NASA INTEX-B mission. Atmos. Chem. Phys..

[28] Dittmar T (2008). The molecular level determination of black carbon in marine dissolved organic matter. Org. Geochem..

[29] Dittmar T (2012). Continuous flux of dissolved black carbon from a vanished tropical forest biome. Nat. Geosci..

[30] Kanakidou M (2012). Atmospheric fluxes of organic N and P to the global ocean. Global Biogeochem. Cycles.

[31] Miyazaki Y, Kawamura K, Jung J, Furutani H, Uematsu M (2011). Latitudinal distributions of organic nitrogen and organic carbon in marine aerosols over the western North Pacific. Atmos. Chem. Phys..

[32] Kirillova EN, Andersson A, Han J, Lee M, Gustafsson Ö (2014). Sources and light absorption of water-soluble organic carbon aerosols in the outflow from northern China. Atmos. Chem. Phys..

[33] Koch BP, Dittmar T (2006). From mass to structure: an aromaticity index for high-resolution mass data of natural organic matter. Rapid Commun. Mass Spectrom..

[34] Kieber DJ (2016). Coupled ocean-atmosphere loss of marine refractory dissolved organic carbon. Geophys. Res. Lett..

[35] Chalbot MC (2013). Soil humic-like organic compounds in prescribed fire emissions using nuclear magnetic resonance spectroscopy. Environ. Pollut..

[36] Mladenov N, López-Ramos J, McKnight DM, Reche I (2009). Alpine lake optical properties as sentinels of dust deposition and global change. Limnol. Oceanogr..

[37] Lin P, Laskin J, Nizkorodov SA, Laskin A (2015). Revealing brown carbon chromophores produced in reactions of methylglyoxal with ammonium sulfate. Environ. Sci. Technol..

[38] Wagner S, Dittmar T, Jaffé R (2015). Molecular characterization of dissolved black nitrogen via electrospray ionization Fourier transform ion cyclotron resonance mass spectrometry. Org. Geochem..

[39] Knicker H (2007). How does fire affect the nature and stability of soil organic nitrogen and carbon? A review. Biogeochemistry.

[40] Wagner S (2015). Linking the molecular signature of heteroatomic dissolved organic matter to watershed characteristics in world rivers. Environ. Sci. Technol..

[41] Kundu S (2013). Evidence and quantitation of aromatic organosulfates in ambient aerosols in Lahore, Pakistan. Atmos. Chem. Phys..

[42] Stubbins A, Niggemann J, Dittmar T (2012). Photo-lability of deep ocean dissolved black carbon. Biogeosciences.

[43] Wilson TW (2015). A marine biogenic source of atmospheric ice-nucleating particles. Nature..

[44] Putman AL (2012). Ultrahigh-resolution FT-ICR mass spectrometry characterization of alpha-pinene ozonolysis SOA. Atmos. Environ..

[45] Whitehouse BG (1984). The effects of temperature and salinity on the aqueous solubility of polynuclear aromatic hydrocarbons. Mar. Chem..

[46] Mulitza S (2010). Increase in African dust flux at the onset of commercial agriculture in the Sahel region. Nature..

[47] Shao Y, Klose M, Wyrwoll KH (2013). Recent global dust trend and connections to climate forcing. J. Geophys. Res. Atmos.

[48] Schlitzer, R. Ocean Data View v. 4.7.10 (2017).

[49] Stafford RG, Ettinger HJ (1972). Filter efficiency as a function of particle size and velocity. Atmos. Environ..

[50] Dittmar T, Koch B, Hertkorn N, Kattner G (2008). A simple and efficient method for the solid-phase extraction of dissolved organic matter (SPE-DOM) from seawater. Limnol. Oceanogr. Methods.

[51] Stubbins A (2015). Utilizing colored dissolved organic matter to derive dissolved black carbon export by arctic rivers. Front. Earth Sci.

[52] Seidel M (2014). Biogeochemistry of dissolved organic matter in an anoxic intertidal creek bank. Geochim. Cosmochim. Acta..

[53] R Core Team. *R: a language and environment for statistical computing*. (R Foundation for Statistical Computing, 2015).

[54] Hoppel WA, Frick GM, Fitzgerald JW (2002). Surface source function for sea-salt aerosol and aerosol dry deposition to the ocean surface. J. Geophys. Res. Atmos.

[55] Gao Y (1997). Temporal and spatial distributions of dust and its deposition to the China Sea. Tellus, Series B: Chem. Phy. Meteorol..

[56] Bao H, Wu Y, Zhang J (2015). Spatial and temporal variation of dissolved organic matter in the Changjiang: fluvial transport and flux estimation. J. Geophys. Res. Biogeosci.

[57] Wang X, Ma H, Li R, Song Z, Wu J (2012). Seasonal fluxes and source variation of organic carbon transported by two major Chinese Rivers: the Yellow River and Changjiang (Yangtze) River. Global Biogeochem. Cycles.

[58] Willey D, Kieber RJ, Eyman MS, Brooks AJG (2000). Rainwater dissolved organic carbon: concentrations and global flux. Global Biogeochem. Cycles.

