# Supplementary material for: Aerosols as a source of dissolved black carbon to the ocean
Source: Nat Commun. 2017 Sep 11;8:510. doi: 10.1038/s41467-017-00437-3 (PMC5593878; doi:10.1038/s41467-017-00437-3)
Supplement: Supplementary file 1 — Supplementary Information [file 41467_2017_437_MOESM1_ESM.pdf]

Title: Supplementary Information

Description: Supplementary Figures, Supplementary Tables, and Supplementary References

Title: Supplementary Data 1

Description: Water soluble organic carbon (WSOC), water soluble black carbon (WSBC) concentrations and WSBC/WSOC ratios of each sample.

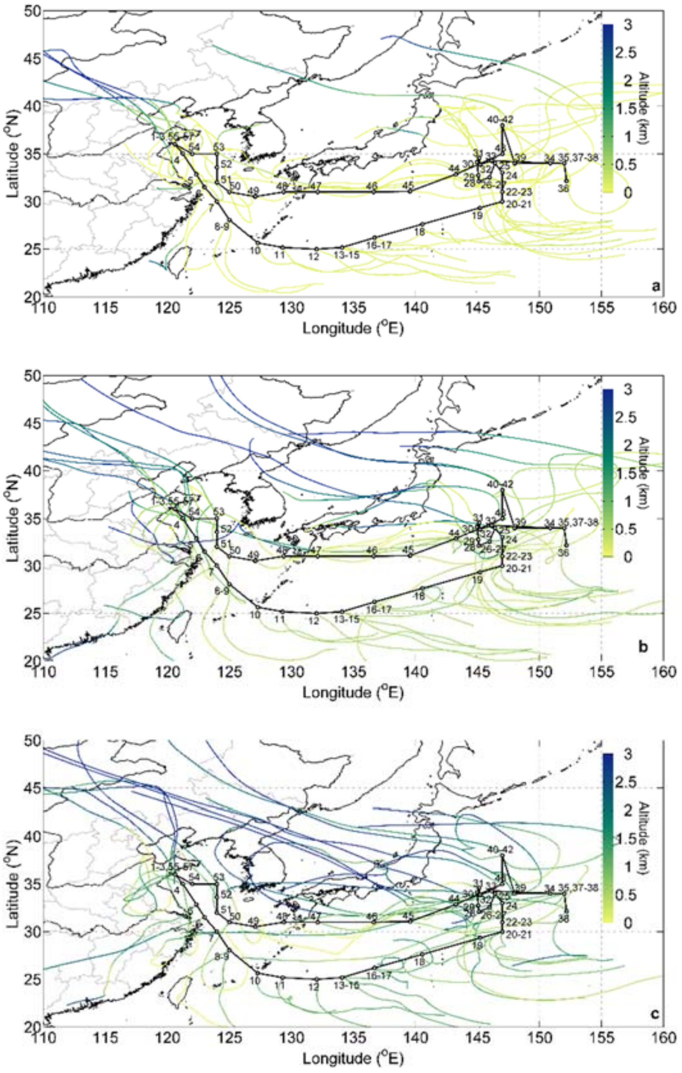

Supplementary Fig. 1: Three-day backward trajectories of all the aerosol samples. Three heights of above-sea-level air mass backward trajectories were calculated using the National Oceanic and Atmospheric Administration (NOAA) Hybrid Single Particle Lagrangian Integrated Trajectory (HYSPLIT) model with a 1°×1° latitude-longitude grid. Details about the HYSPLIT model can be found at http://www.arl.noaa.gov/HYSPLIT_info.php . The time period of 3 days was suggested to be sufficient for dust transport from dust sources to the northwestern Pacific Ocean [1].

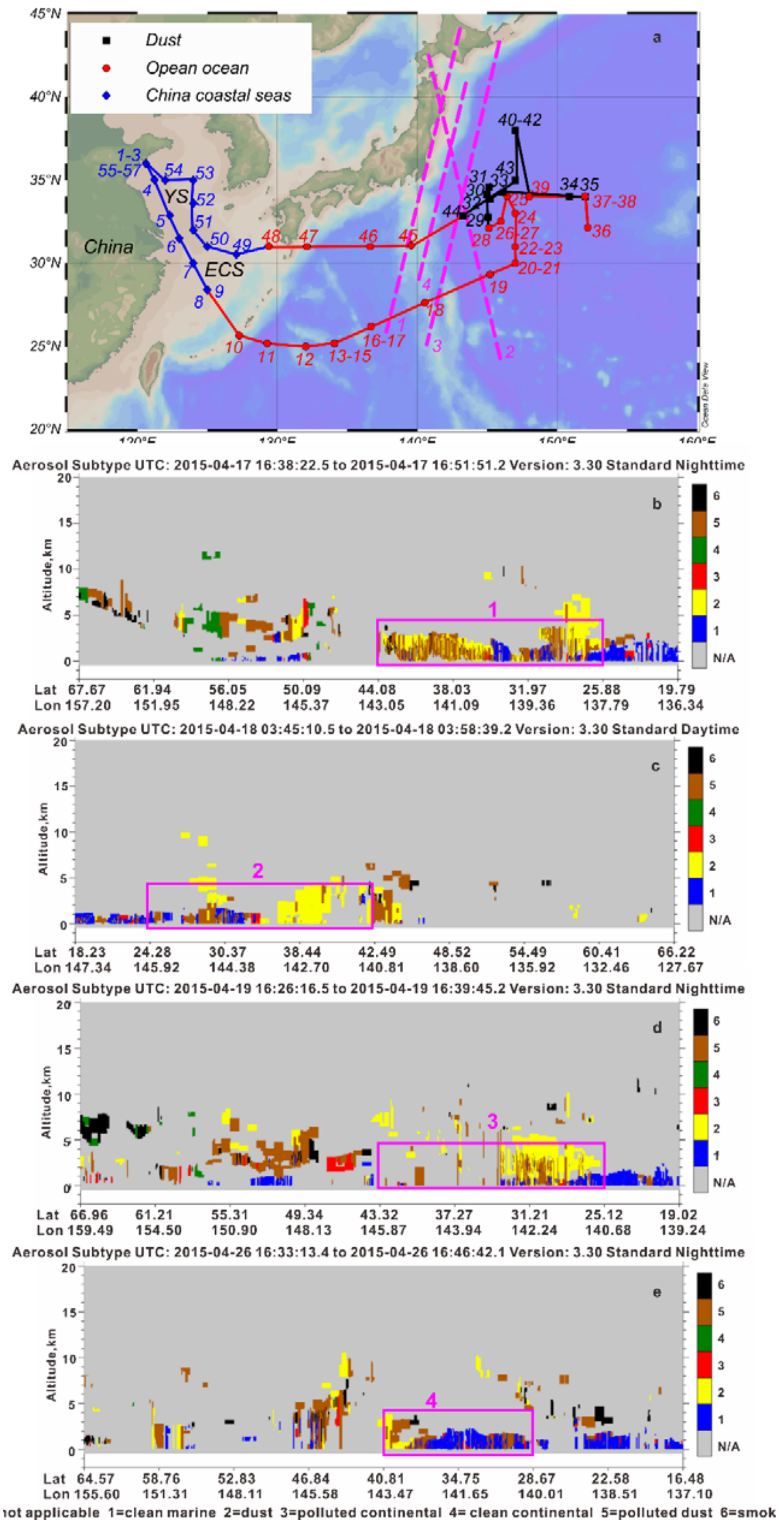

Supplementary Fig. 2: NASA satellite images showing the dust effect during our sampling.

Extension of the four transects in a) is indicated on the map b, c, d and e. NASA satellite images were obtained from https://www-calipso.larc.nasa.gov/.

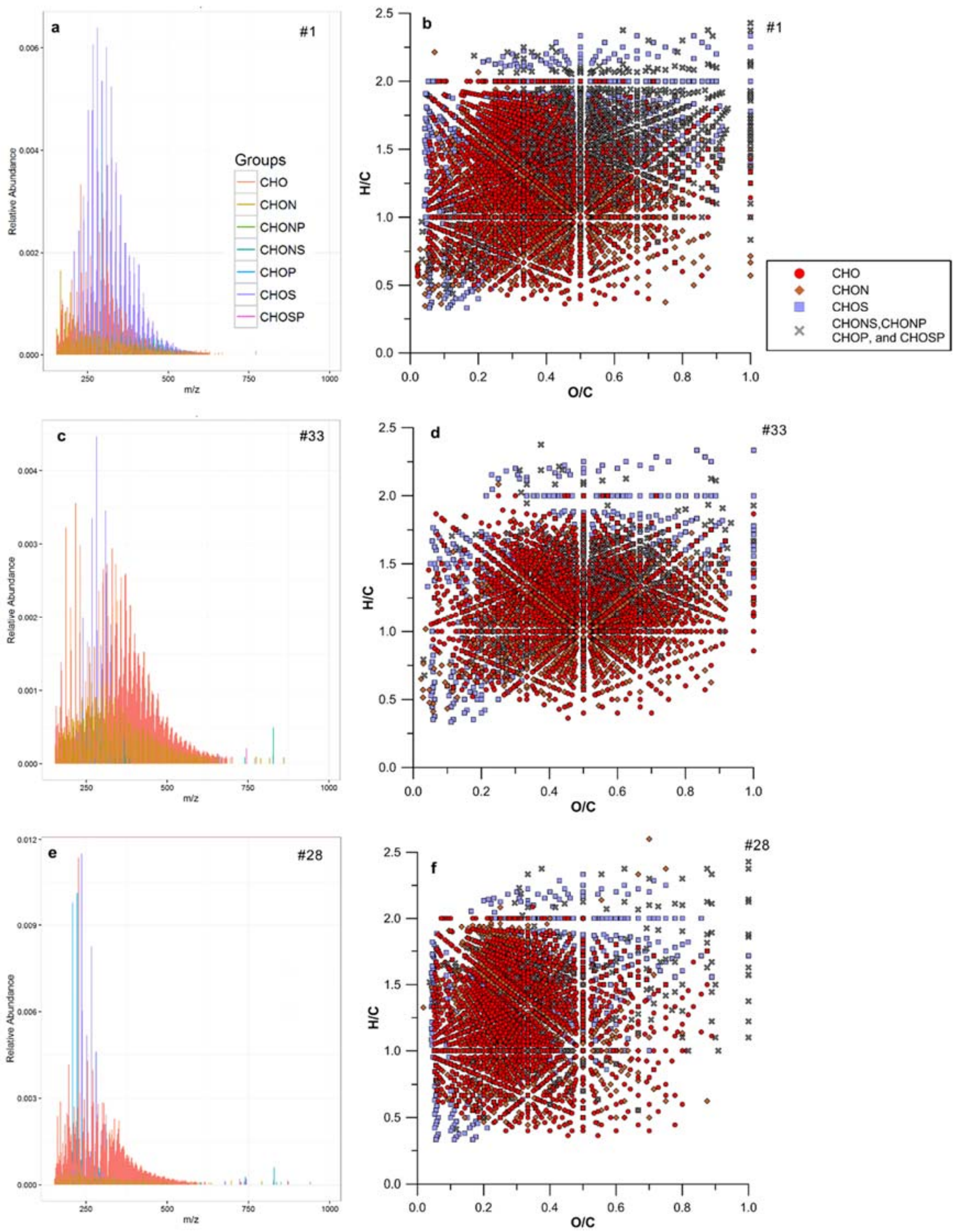

Supplementary Fig. 3: Reconstructed mass spectra (a, c and e) and van Krevelen diagrams (b, d and f) of example samples #1 (China coastal seas), #33 (dust) and #28 (Open ocean).

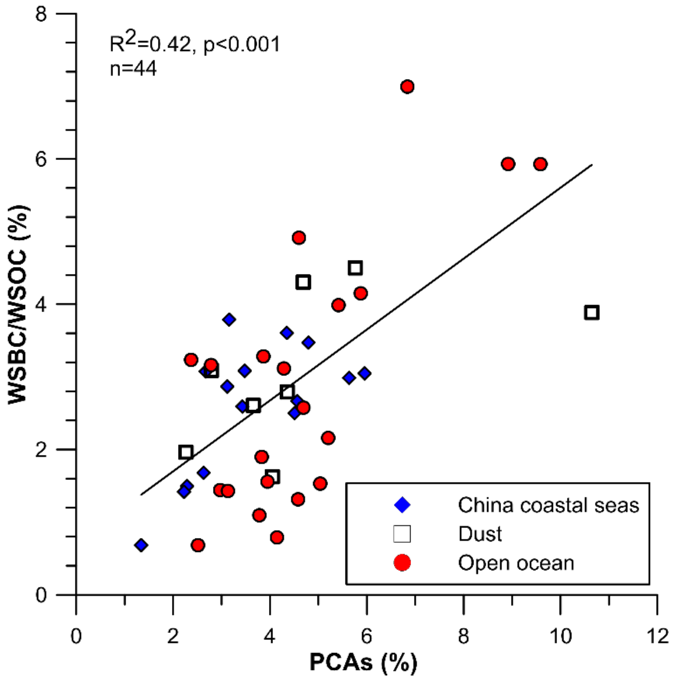

Supplementary Fig. 4: Correlation between the relative signal intensity of combustion-derived polycyclic aromatic compounds (PCAs) determined by Fourier transform ion cyclotron resonance mass spectrometry (FT-ICR-MS) and water soluble black carbon (WSBC) to water soluble organic carbon (WSOC) (WSBC/WSOC) ratios.

Supplementary Table 1: Summary of general molecular characteristics of different groups of aerosols. Numbers in brackets represent average value.

	China coastal seas (n=15)	Dust (n=9)	Open ocean (n=22)
Number of formulas	4285 – 6678 (5654)	4347 – 5574 (4961)	2984 – 6780 (3976)
Mass	317 – 358 (340)	337 – 393 (361)	316 – 362 (334)
AI_{mod}	0.13 – 0.23 (0.18)	0.18 – 0.33 (0.24)	0.15 – 0.32 (0.23)
H/C	1.4 – 1.6 (1.4)	1.2 – 1.3 (1.3)	1.2 – 1.6 (1.4)
O/C	0.42 – 0.53 (0.46)	0.37 – 0.56 (0.48)	0.31 – 0.51 (1.37)
CHO	30 – 55% (43%)	47 – 67% (56%)	43 – 67% (57%)
CHON	16 – 28% (21%)	17 – 35% (28%)	12 – 34% (23%)
CHOS	22 – 42% (32%)	7.1 – 23% (14%)	5.9 – 36% (16%)
CHOP	0.29 – 1.3% (0.72%)	0.30 – 2.0% (1.1%)	0.8 –2.7% (1.4%)
CHONS	0.97 – 6.2% (2.9%)	0.16 – 1.4% (0.64%)	0.31 – 1.3% (0.80%)
CHONP	<0.1%	<0.1%	<0.1 – 0.36% (0.15%)
CHOSP	0.10 – 0.80% (0.32%)	0.10 – 0.57% (0.28%)	0.24 – 1.1% (0.61%)

Supplementary Table 2: Fraction of polycyclic aromatic compounds (PCAs) compounds (percentage of total intensity) in different samples, intensity normalized parameters and contribution of different PCAs compounds to all PCAs in the respective sample. Numbers in brackets represent average value.

	China coastal seas (n=15)	Dust (n=9)	Open ocean (n=22)
PCAs (% of total intensity)	1.3 – 5.9 (3.9)	2.3 – 11 (4.5)	2.3 – 9.6 (4.5)
H/C (PCAs)	0.58 – 0.62 (0.60)	0.59 – 0.60 (0.59)	0.58 – 0.60 (0.59)
O/C (PCAs)	0.23 – 0.35 (0.30)	0.29 – 0.37 (0.33)	0.26 – 0.37 (0.30)
PCAas (% of PCAs)	38 – 58% (46%)	41 – 49% (44%)	35 – 53% (46%)
PCAar (% of PCAs)	72 – 92% (86%)	79 – 93% (86%)	73 – 93% (85%)
AI_{mod}as	0.71 – 0.73 (0.72)	0.72 (0.72)	0.71 – 0.73 (0.72)
AI_{mod}ar	0.74 – 0.75 (0.75)	0.74 – 0.75 (0.75)	0.74 – 0.76 (0.75)
Mass_as	235 – 258 (246)	239 – 245 (242)	226 – 254 (239)
Mass_ar	243 – 268 (254)	241 – 255 (248)	231 – 258 (244)
H/Cas	0.63 – 0.66 (0.64)	0.62 – 0.64 (0.64)	0.63 – 0.65 (0.64)
O/Cas	0.23 – 0.32 (0.28)	0.26 – 0.34 (0.30)	0.25 – 0.35 (0.28)
H/Car	0.57 – 0.61 (0.58)	0.56 – 0.58 (0.58)	0.57 – 0.58 (0.57)
O/Car	0.34 – 0.43 (0.36)	0.33 – 0.36 (0.35)	0.32 – 0.37 (0.35)
CHO (% of PCAs)	30 – 61 (44)	28 – 70 (48)	20 – 73 (46)
CHON (% of PCAs)	20 – 45 (31)	19 – 41 (31)	16 – 36 (25)
CHOS (% of PCAs)	7 – 36 (22)	7 – 40 (19)	10 – 47 (25)
Others (% of PCAs)	0.72 – 6.6 (2.6)	0.25 – 5 (2.2)	0.76 – 9.6 (3.2)

References

1. Husar, R. B. *et al.* Asian dust events of April 1998. *J. Geophys. Res. Atmos.* **106,** 18317–18330 (2001).
